# Supplementary material for: Sulfur oxidation and reduction are coupled to nitrogen fixation in the roots of the salt marsh foundation plant Spartina alterniflora
Source: Nat Commun. 2024 Apr 29;15:3607. doi: 10.1038/s41467-024-47646-1 (PMC11059160; doi:10.1038/s41467-024-47646-1)
Supplement: Supplementary file 1 — Supplementary Information [file 41467_2024_47646_MOESM1_ESM.pdf]

SUPPLEMENTARY INFORMATION

Sulfur oxidation and reduction are coupled to nitrogen fixation in the roots of the salt marsh foundation plant *Spartina alterniflora*

Authors: Rolando, J.L.[a]; Kolton, M.[a,b]; Song, T.[a], Liu, Y.[a,c]; Pinamang, P.[a], Conrad, R.[a]; Morris, J.T.[d], Konstantinidis, K.T.[a,e], Kostka, J.E. [a,f,g]

Affiliations:

[a]Georgia Institute of Technology, School of Biological Sciences, Atlanta, GA 30332, USA

[b]French Associates Institute for Agriculture and Biotechnology of Drylands, Ben-Gurion University of the Negev, Beer Sheva, Israel

[c]The Pennsylvania State University, Department of Civil & Environmental Engineering, University Park, PA, 16802

[d] Belle Baruch Institute for Marine & Coastal Sciences, University of South Carolina, Columbia, SC, 29201

[e]Georgia Institute of Technology, School of Civil and Environmental Engineering, Atlanta, GA 30332, USA

[f]Georgia Institute of Technology, School of Earth and Atmospheric Sciences, Atlanta, GA 30332, USA

[g]Center for Microbial Dynamics and Infection, Georgia Institute of Technology, Atlanta, GA 30332, USA

Corresponding author:

Kostka, JE. e-mail: joel.kostka@biology.gatech.edu

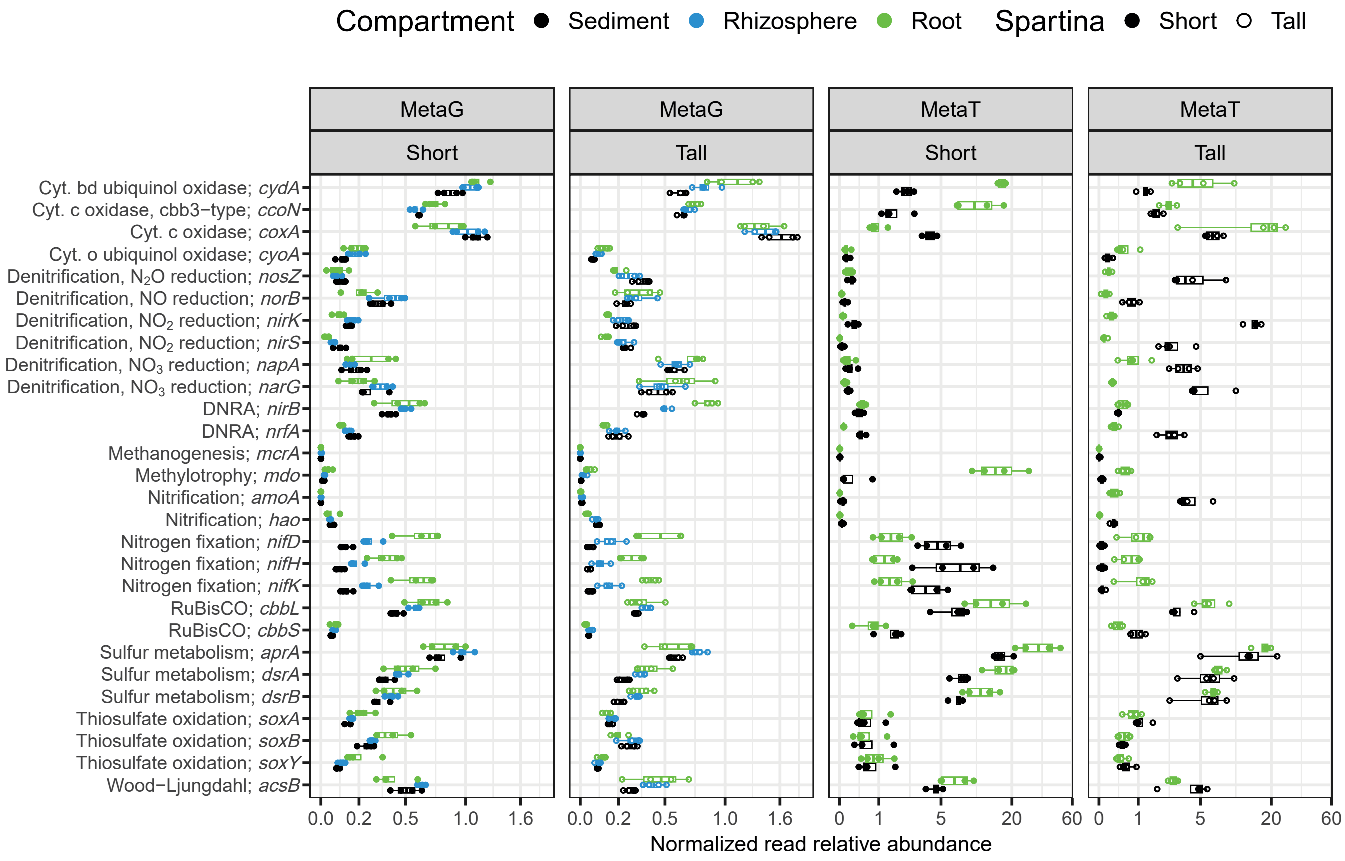

Supplementary Fig. S1. Gene and transcript normalized relative abundance of prokaryotic terminal oxidases and selected enzymes of the carbon, nitrogen, and sulfur cycles (n = 4). In boxplots, boxes are defined by the upper and lower interquartile; the median is represented as a horizontal line within the boxes; whiskers extend to the most extreme data point which is no more than 1.5 times the interquartile range

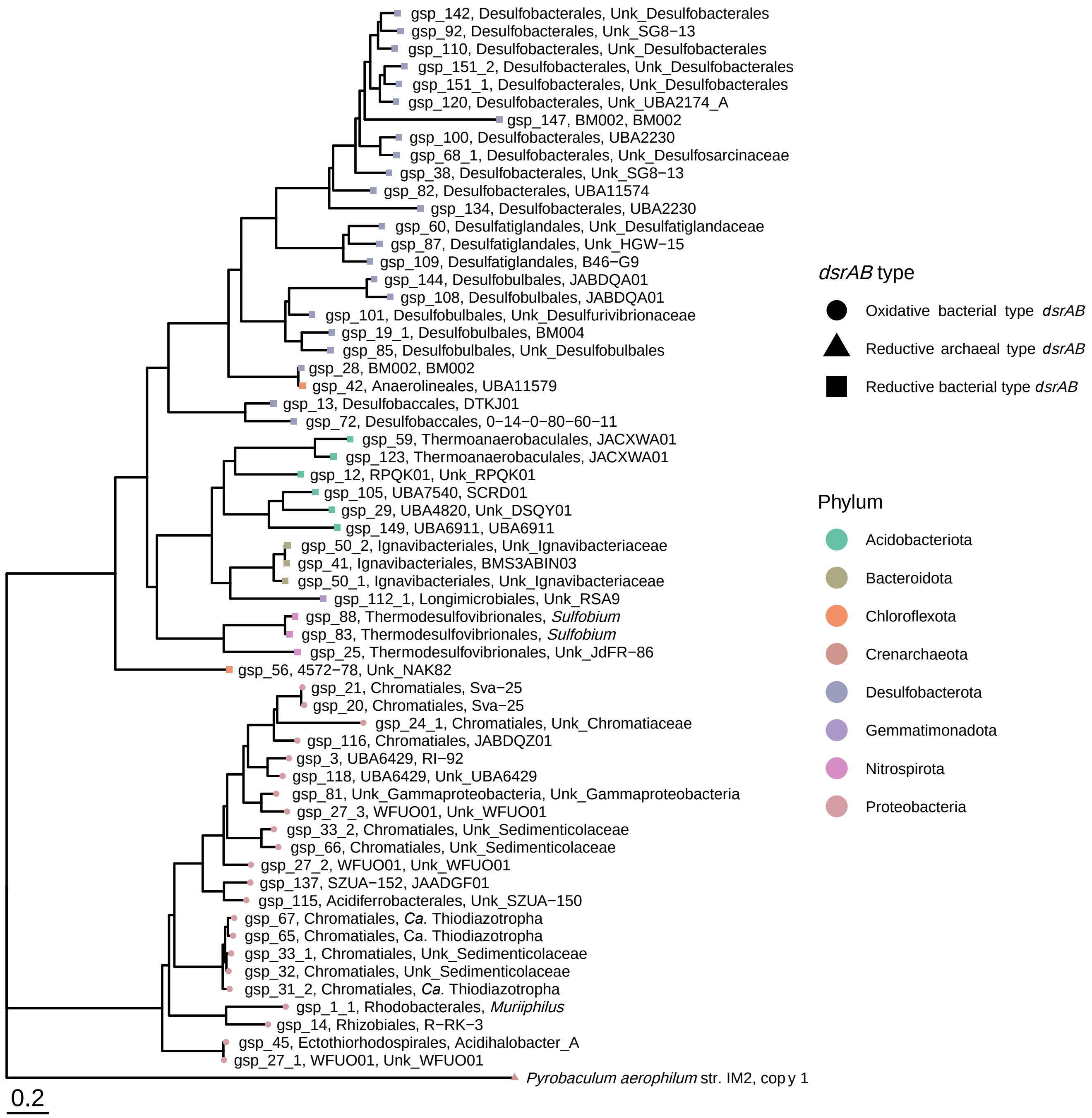

Supplementary Fig. S2. *dsrAB* phylogenetic tree of genes retrieved from metagenome-assembled genomes (MAGs). Genes were aligned to a reference database and a phylogenetic tree was constructed with FastTree v.2.1.11. Oxidative and reductive types of the *dsrAB* gene were inferred based on phylogenetic placement in the constructed tree. Archean *Pyrobaculum aerophilum* was used as an outgroup.

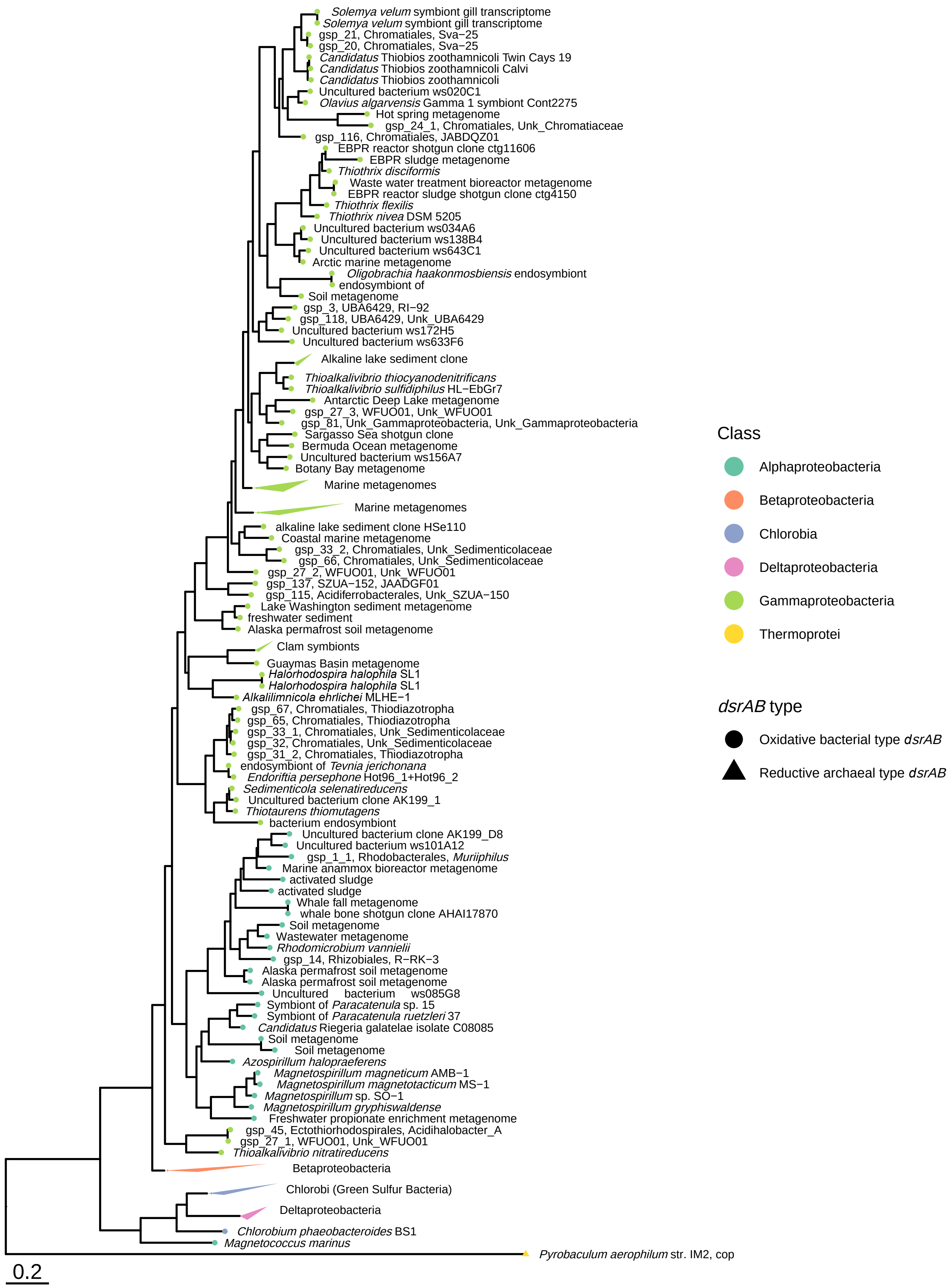

Supplementary Fig. S3. Phylogenetic tree of the oxidative *dsrAB* type from metagenome assembled genomes (MAGs) aligned to a reference alignment database (Müller et al., 2015). Archean *Pyrobaculum aerophilum* was used as an outgroup of the phylogenetic tree.

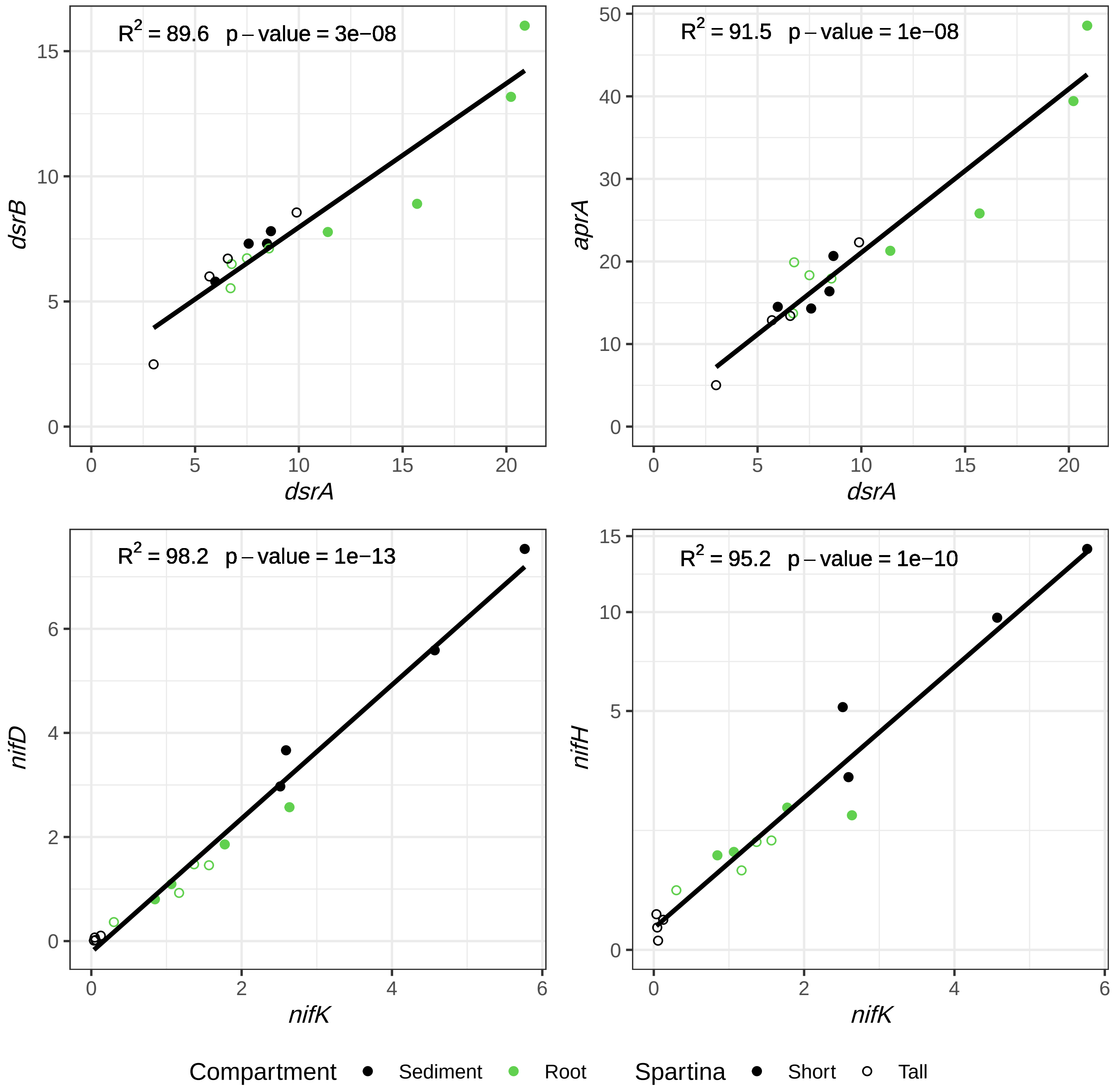

Supplementary Fig. S4. Gene expression of the nitrogenase (*nifD*, *nifK*, and *nifH*) and dissimilatory sulfite reductase genes (*dsrA*, *dsrB*, and *aprA*) are highly correlated among their own metabolic pathways. Gene expression is quantified as the normalized transcript counts per metatranscriptomic sample.

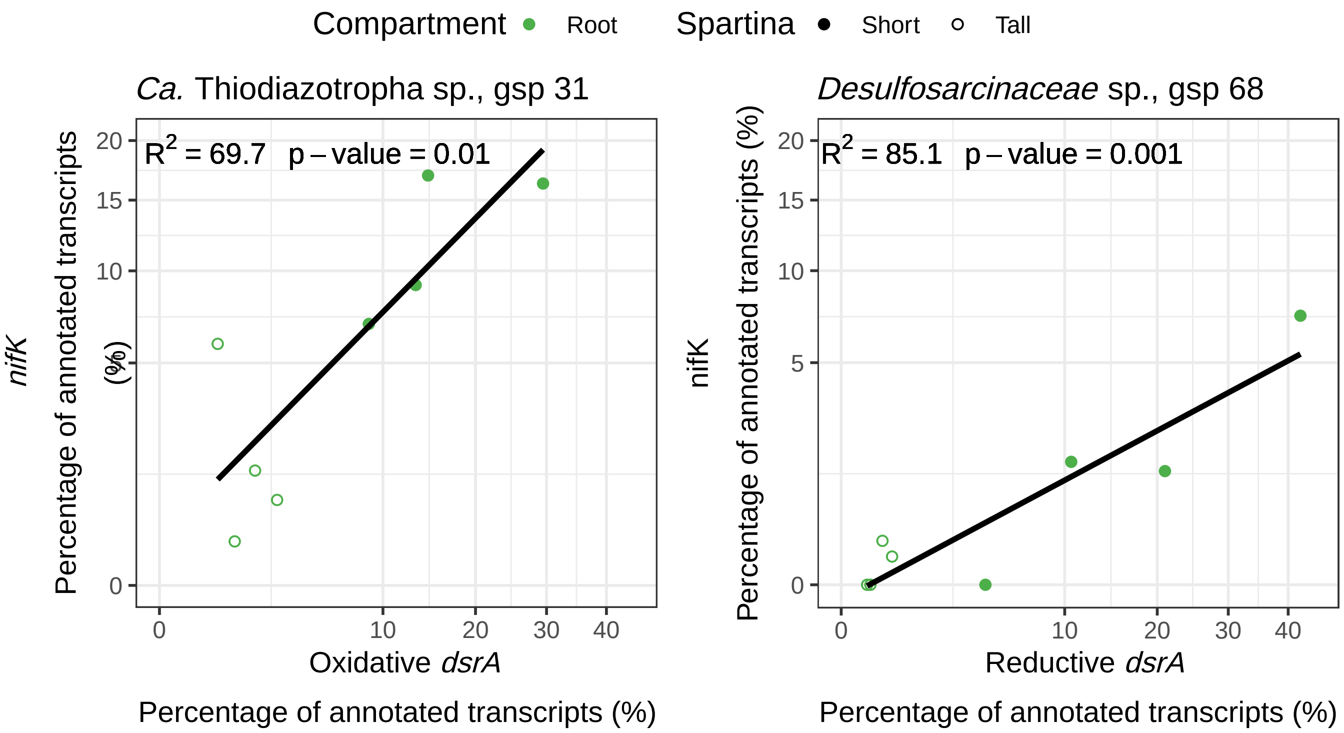

Supplementary Fig. S5. Relation between the gene expression of the *Ca.* Thiodiazotropha sp. (gsp 31) oxidative, and *Desulfosarcinaceae* sp. (gsp 68) reductive *dsrA* gene and the nitrogenase gene *nifK* in *Spartina alterniflora* root samples. Gene expression measured as the percentage of transcripts mapping to each genomospecies from the totality of functionally annotated short read transcripts (community level).

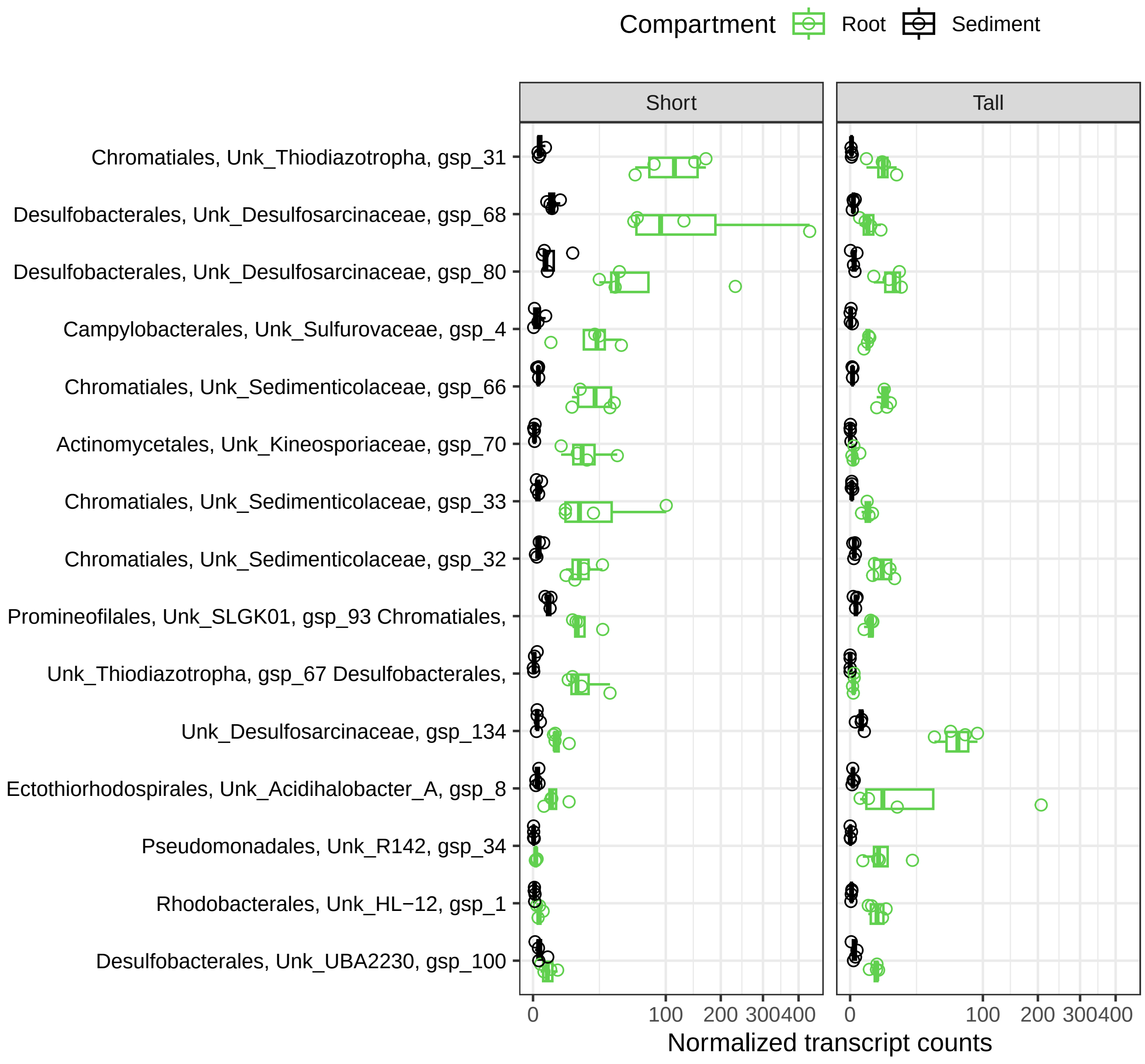

Supplementary Fig. S6. Normalized transcript counts of the 15 most active genomospecies in the root compartment. Taxonomy information is presented as classified by gtdb-tk, using reference database R07-RS207. Gsp 66, gsp 33, and gsp 32 are proposed to be member of the *Candidatus* Thiodiazotropha genus based on phylogenetic analysis (Figure 5). In boxplots, boxes are defined by the upper and lower interquartile; the median is represented as a horizontal line within the boxes; whiskers extend to the most extreme data point which is no more than 1.5 times the interquartile range.

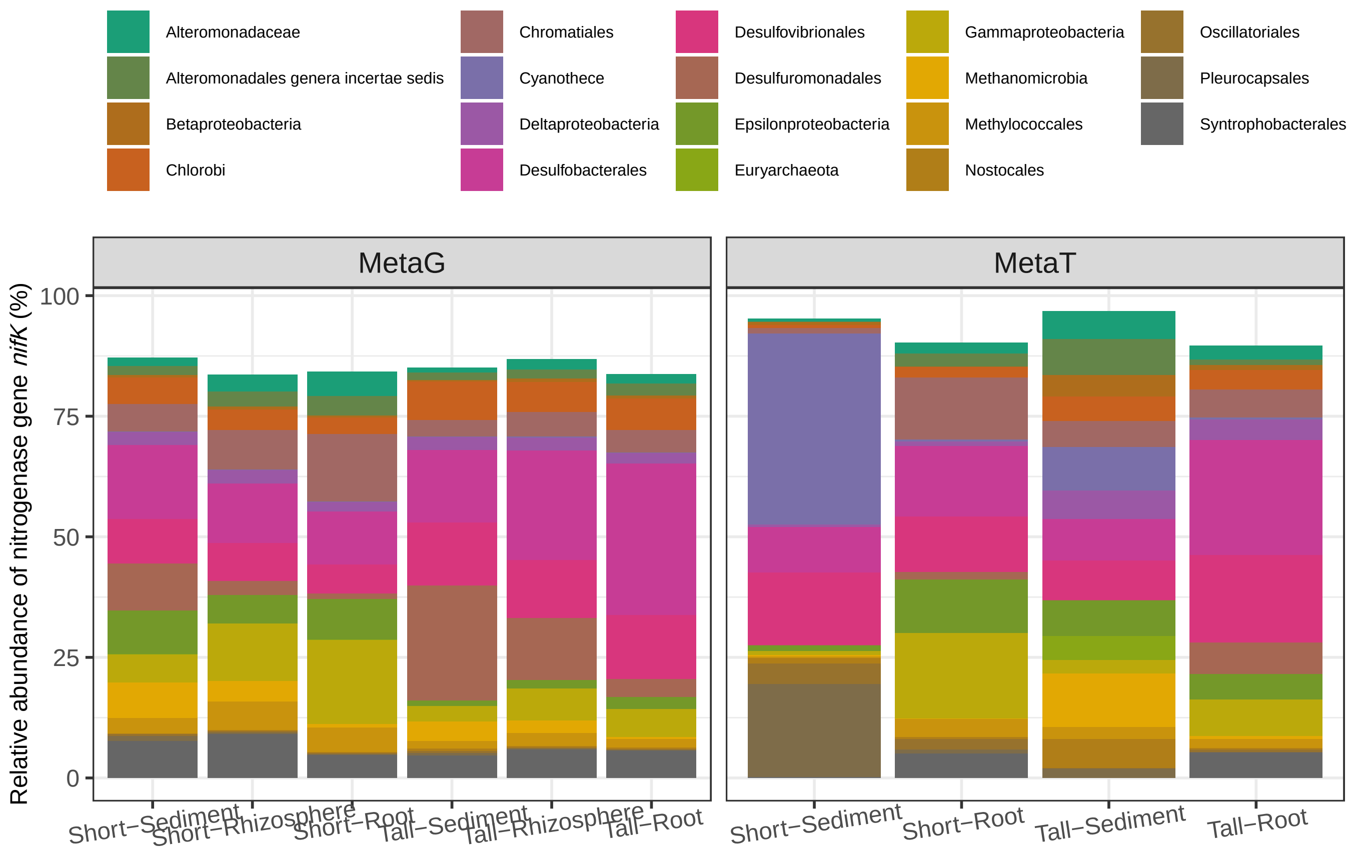

Supplementary Fig. S7. Mean gene and transcript relative abundance of the nitrogenase gene (*nifK*) partitioned at the finest taxonomic level as predicted by eggnog-mapper per microbiome compartment and *Spartina alterniflora* phenotype.

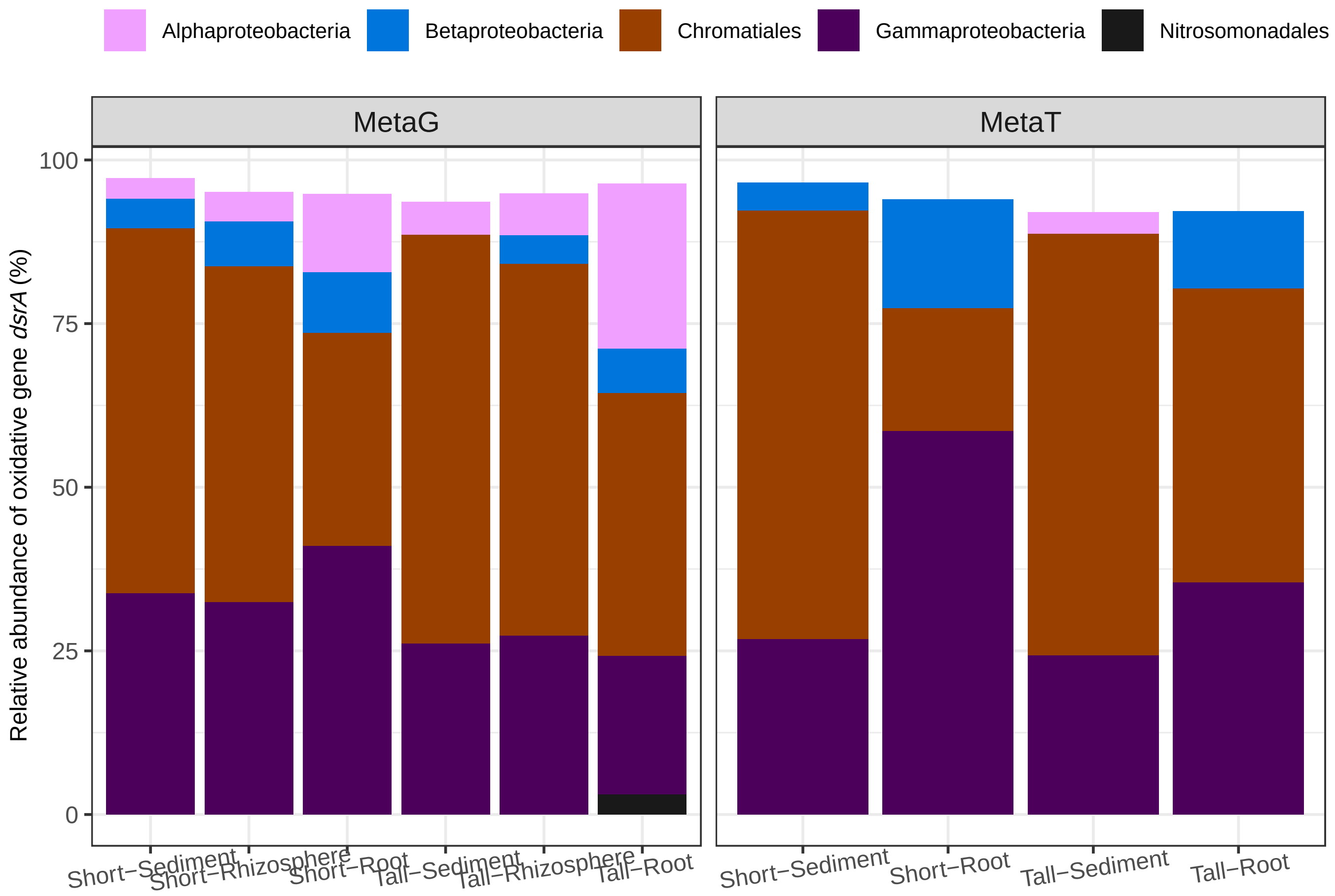

Supplementary Fig. S8. Mean gene and transcript relative abundance of the oxidative *dsrA* gene partitioned at the finest taxonomic level as predicted by eggnog-mapper per microbiome compartment and *Spartina alterniflora* phenotype.

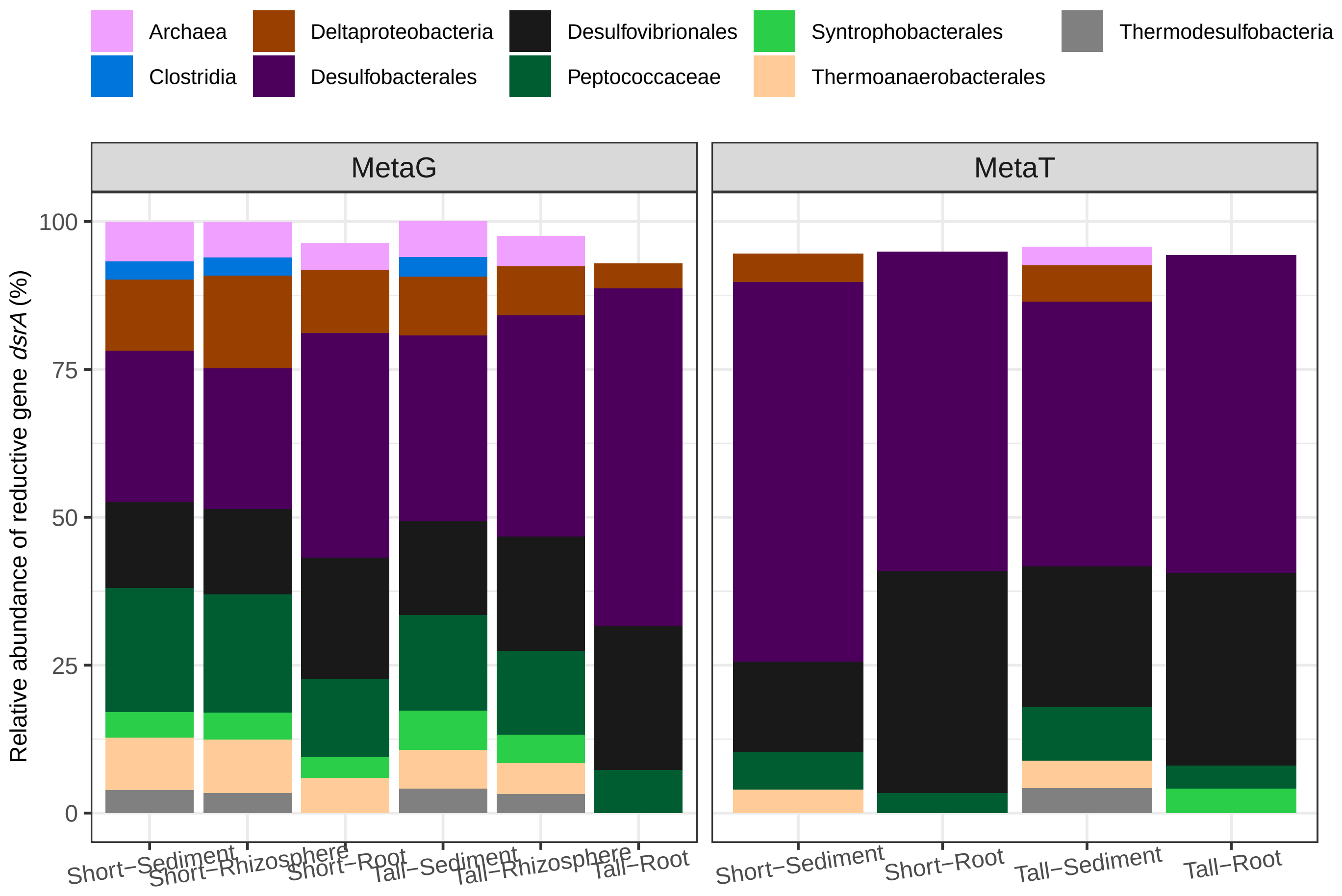

Supplementary Fig. S9. Gene and transcript relative abundance of the reductive *dsrA* gene partitioned at the finest taxonomic level as predicted by eggnog-mapper per microbiome compartment and *Spartina alterniflora* phenotype.

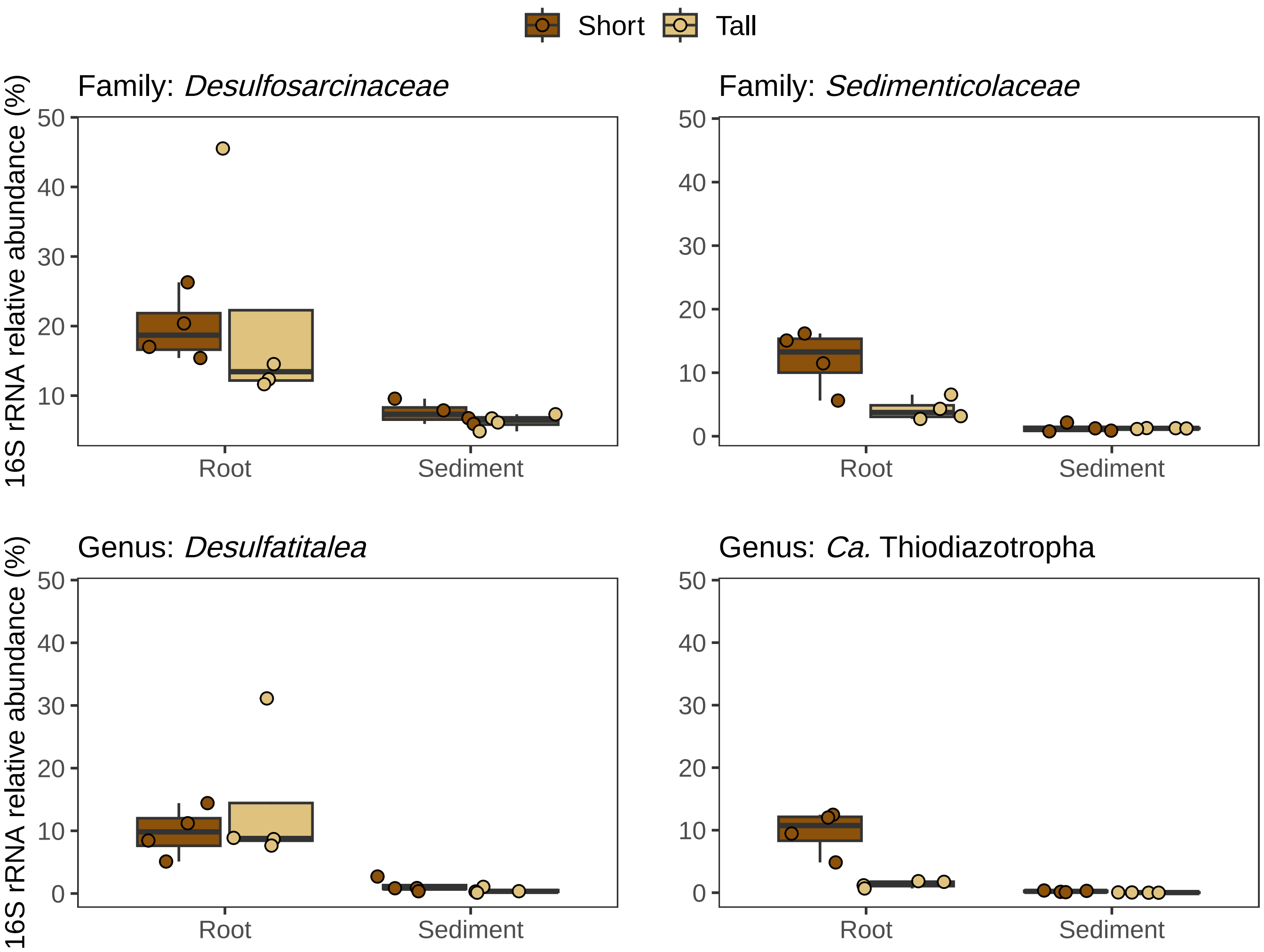

Supplementary Fig. S10. Relative abundance of 16S rRNA amplicon sequence variants (ASVs) classified as members of the *Desulfosarcinaceae* and *Sedimenticolaceae* families, and the *Desulfatitalea* and *Ca.* Thiodiazotropha genera across sediment and root samples from the short and tall *Spartina alterniflora* phenotypes (n = 4). In boxplots, boxes are defined by the upper and lower interquartile; the median is represented as a horizontal line within the boxes; whiskers extend to the most extreme data point which is no more than 1.5 times the interquartile range

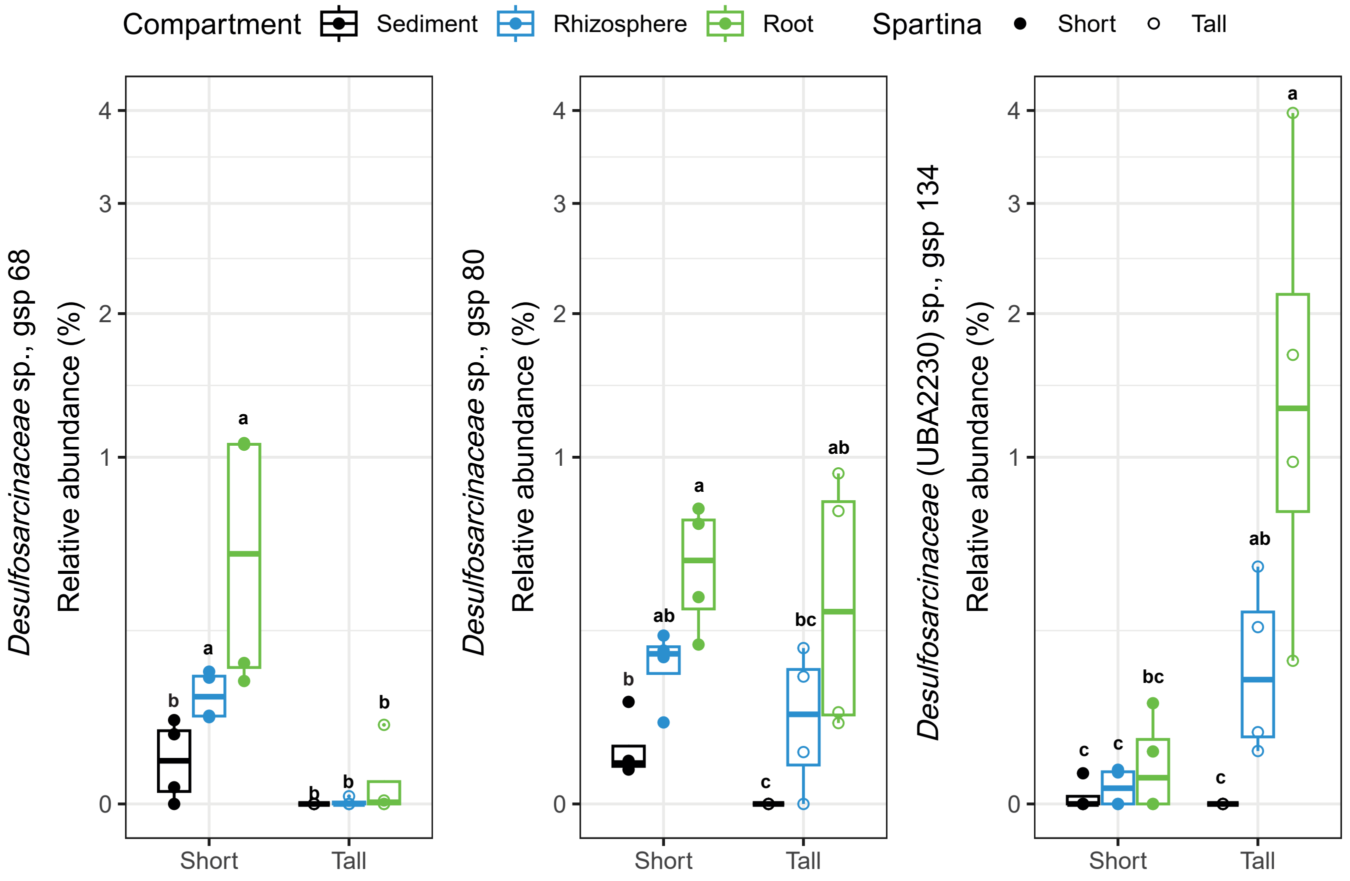

Supplementary Fig. S11. Relative abundance of selected metagenome assembled genomes (MAGs) from the *Desulfosarcinaceae* family across *Spartina alterniflora* phenotype and sampled compartment (n = 4, per phenotype and compartment). Relative abundance was calculated at the DNA-level based on average coverage per position in metagenomic libraries. In boxplots, boxes are defined by the upper and lower interquartile; the median is represented as a horizontal line within the boxes; whiskers extend to the most extreme data point which is no more than 1.5 times the interquartile range. Different letter indicates statistical difference based on pairwise Mann-Whitney tests (p-value < 0.05, two-sided).

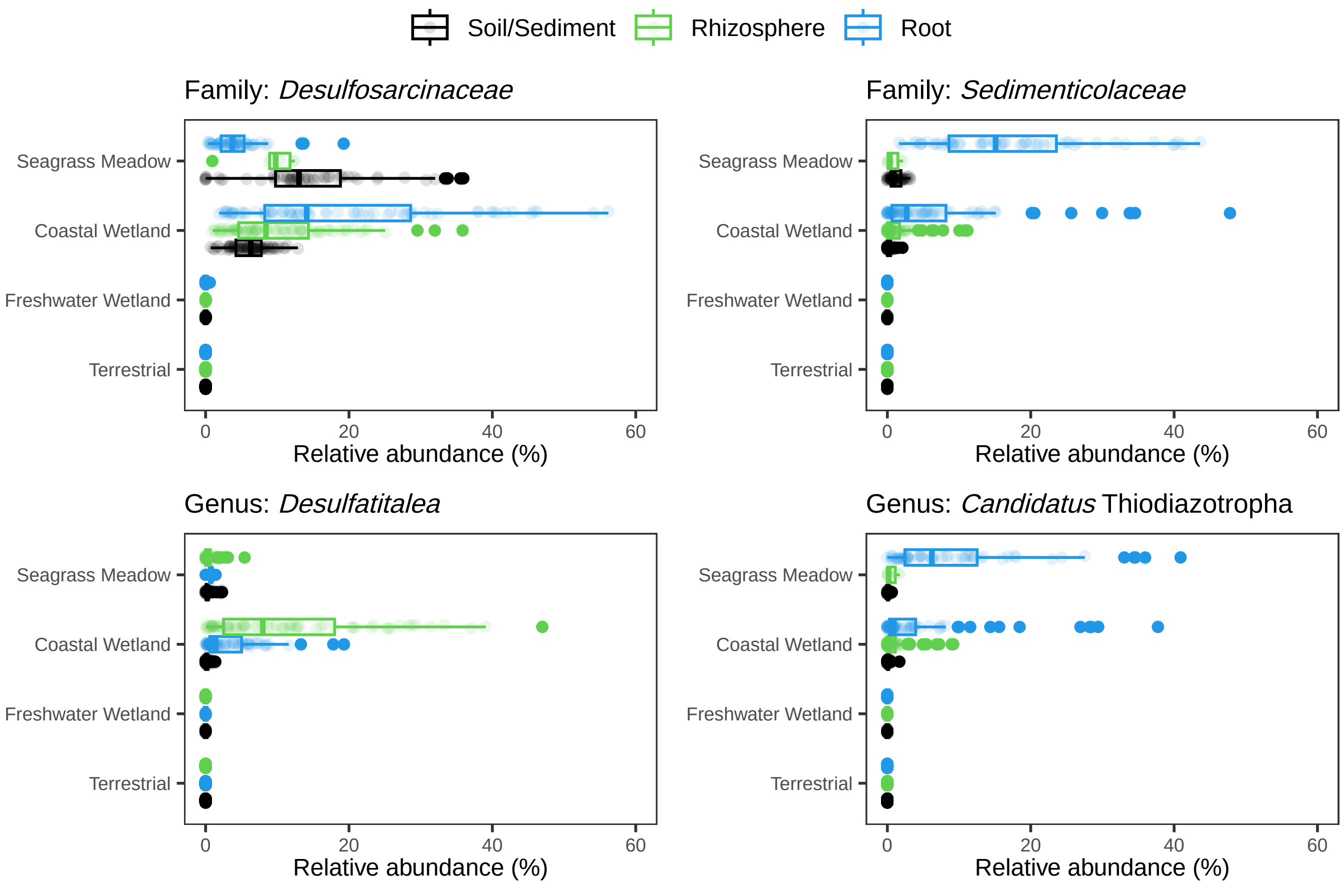

Supplementary Fig. S12. Relative abundance of 16S rRNA gene amplicon sequence variants (ASVs) classified as members of the *Desulfosarcinaceae* and *Sedimenticolaceae* families, and the *Desulfatitalea* and *Ca.* Thiodiazotropha genera across soil/sediment, rhizosphere, and root samples from a seagrass meadows, coastal wetland, freshwater wetland and terrestrial ecosystems dataset. In boxplots, boxes are defined by the upper and lower interquartile; the median is represented as a horizontal line within the boxes; whiskers extend to the most extreme data point which is no more than 1.5 times the interquartile range.

Supplementary Table S1: Analysis of the deterministic parameters controlling metagenomic and metatranscriptomic functional profile by compartment and *Spartina alterniflora* phenotype. PERMANOVA analysis was conducted using the Bray-Curtis metric with 999 permutations using matrices of normalized KEGG KO counts.

Library	Factor	Degrees of freedom	SumsOfSqs	MeanSqs	F.Model	R^2	Pr(>F)
Metagenome	Compartment	1	0.05	0.052	15.5	0.254	0.001
	Spartina phenotype	2	0.08	0.042	12.8	0.419	0.001
	Residuals	20	0.07	0.003	0.3		
	Total	23	0.20	1.000			
Metatranscriptome	Compartment	1	0.11	0.11	5.5	0.158	0.006
	Spartina phenotype	1	0.32	0.32	16.2	0.467	0.001
	Residuals	13	0.26	0.02	0.4		
	Total	15	0.69	1.00			

Supplementary Table S2: Analysis of the deterministic parameters controlling root microbiome assembly across ecosystem types and compartment. PERMANOVA analysis was conducted using the Bray-Curtis metric with 999 permutations. Based on 2911 16S rRNA amplicon libraries from root microbiomes.

Factor	Degrees of freedom	SumsOfSqs	MeanSqs	F.Model	R^2	Pr(>F)
Compartment	2	64.0	32.0	112.64	0.062	0.001
Ecosystem type	3	135.5	45.2	159.01	0.132	0.001
Residuals	2905	825.0	0.3	0.80531		
Total	2910	1024.4	1.0			
