# Supplementary material for: Sulfur oxidation and reduction are coupled to nitrogen fixation in the roots of the salt marsh foundation plant Spartina alterniflora
Source: Nat Commun. 2024 Apr 29;15:3607. doi: 10.1038/s41467-024-47646-1 (PMC11059160; doi:10.1038/s41467-024-47646-1)
Supplement: Supplementary file 3 — Description of Additional Supplementary Files [file 41467_2024_47646_MOESM3_ESM.pdf]

## Description of Additional Supplementary Files

**File name: Supplementary Data File S1.**

**Description:** Sequencing effort and quality of shotgun metagenome and metatranscriptome samples.

**File name: Supplementary Data File S2.**

**Description:** Taxonomic identity and statistics of binned metagenome-assembled genomes (MAGs).

**File name: Supplementary Data File S3.**

**Description:** Copy number of selected genes in the carbon, nitrogen, and sulfur cycles, and terminal oxidases from all metagenome assembled genomes (MAGs).

**File name: Supplementary Data File S4.**

**Description:** Mean normalized gene expression of predicted genes from genomospecies 31 (*Ca.* Thiodiazotropha sp.) by *Spartina alterniflora* phenotype and compartment.

**File name: Supplementary Data File S5.**

**Description:** Mean normalized gene expression of predicted genes from genomospecies 33 (*Sedimenticolaceae* sp.) by *Spartina alterniflora* phenotype and compartment.

**File name: Supplementary Data File S6.**

**Description:** Mean normalized gene expression of predicted genes from genomospecies 68 (*Desulfosarcinaceae* sp.) by *Spartina alterniflora* phenotype and compartment.

**File name: Supplementary Data File S7.**

**Description:** Mean normalized gene expression of predicted genes from genomospecies 134 (*Desulfosarcinaceae* sp.) by *Spartina alterniflora* phenotype and compartment.

**File name: Supplementary Data File S8.**

**Description:** Amplicon Supplementary Dataset of prokaryotic small subunit rRNA from plant root and paired soil/sediment and rhizosphere samples.

**File name: Supplementary Data File S9.**

**Description:** List of genera containing species with known sulfur/sulfate reducing and sulfur oxidizing metabolism.

**File name: Supplementary Data File S10.**

**Description:** Source and taxonomic identity of genomes used for phylogenetic analysis of the *Sedimenticolaceae* family
