# Supplementary material for: Sulfur oxidation and reduction are coupled to nitrogen fixation in the roots of the salt marsh foundation plant Spartina alterniflora
Source: Nat Commun. 2024 Apr 29;15:3607. doi: 10.1038/s41467-024-47646-1 (PMC11059160; doi:10.1038/s41467-024-47646-1)
Supplement: Supplementary file 5 — Reporting Summary [file 41467_2024_47646_MOESM5_ESM.pdf]

nature portfolio

Corresponding author(s): Joel E. Kostka

Last updated by author(s): Mar 23, 2024

Reporting Summary

Nature Portfolio wishes to improve the reproducibility of the work that we publish. This form provides structure for consistency and transparency in reporting. For further information on Nature Portfolio policies, see our Editorial Policies and the Editorial Policy Checklist.

Statistics

For all statistical analyses, confirm that the following items are present in the figure legend, table legend, main text, or Methods section.

n/a	Confirmed	
☐	☒	The exact sample size (*n*) for each experimental group/condition, given as a discrete number and unit of measurement
☐	☒	A statement on whether measurements were taken from distinct samples or whether the same sample was measured repeatedly
☐	☒	The statistical test(s) used AND whether they are one- or two-sided<br>*Only common tests should be described solely by name; describe more complex techniques in the Methods section.*
☒	☐	A description of all covariates tested
☒	☐	A description of any assumptions or corrections, such as tests of normality and adjustment for multiple comparisons
☐	☒	A full description of the statistical parameters including central tendency (e.g. means) or other basic estimates (e.g. regression coefficient) AND variation (e.g. standard deviation) or associated estimates of uncertainty (e.g. confidence intervals)
☐	☒	For null hypothesis testing, the test statistic (e.g. *F*, *t*, *r*) with confidence intervals, effect sizes, degrees of freedom and *P* value noted<br>*Give P values as exact values whenever suitable.*
☒	☐	For Bayesian analysis, information on the choice of priors and Markov chain Monte Carlo settings
☒	☐	For hierarchical and complex designs, identification of the appropriate level for tests and full reporting of outcomes
☒	☐	Estimates of effect sizes (e.g. Cohen's *d*, Pearson's *r*), indicating how they were calculated

Our web collection on statistics for biologists contains articles on many of the points above.

Software and code

Policy information about availability of computer code

Data collection	No software was used for data collection
Data analysis	All software used in the present study is open-source. The following programs were used, with paramenters and usage included in the material and methods sections:<br>BBTools v.38.84<br>FastQC v.0.11.9<br>bowtie2 v.2.4.2<br>samtools v.1.9<br>sortMeRNA v.4.3.4<br>eggnog-mapper v.2.1.9<br>vegan v. 2.5.7<br>nonpareil v. 3.401<br>idba-ud v.1.1.3<br>MaxBin v.2.2.7<br>MetaBAT v.2.15<br>CONCOCT v.1.1.0<br>DAS Tool v.1.1.2<br>MAGPurify v.2.1.2<br>MiGA v. 0.7<br>Prodigal v.2.6.3<br>bedtools v.2.29.2

enveomics collection
MicrobeCensus v.1.1.0
GTDB-tk v.2.1.0
PhyloPhlAn v3.0.58
Megablast v.2.10.1
Clustal Omega v.1.2.4
FastTree v.2.1.11
Cutadapt v.3.7
DADA2 v.1.20
Phyloseq v.1.36
R 4.1.0
Custom R scripts are available in a Zenodo repository:
https://doi.org/10.5281/zenodo.7883423

For manuscripts utilizing custom algorithms or software that are central to the research but not yet described in published literature, software must be made available to editors and reviewers. We strongly encourage code deposition in a community repository (e.g. GitHub). See the Nature Portfolio guidelines for submitting code & software for further information.

Data

Policy information about availability of data

All manuscripts must include a data availability statement. This statement should provide the following information, where applicable:

- Accession codes, unique identifiers, or web links for publicly available datasets
- A description of any restrictions on data availability
- For clinical datasets or third party data, please ensure that the statement adheres to our policy

The raw metagenomic and metatranscriptomic sequences generated in this study have been deposited in the BioProject database (http://ncbi.nlm.nih.gov/bioproject) under accession codes PRJNA703972 (https://www.ncbi.nlm.nih.gov/bioproject/PRJNA703972/) and PRJNA950121 (https://www.ncbi.nlm.nih.gov/bioproject/PRJNA950121/), respectively. The metagenome-assembled genomes generated in this study have been deposited in the BioProject database under accession code PRJNA703972 (https://www.ncbi.nlm.nih.gov/bioproject/PRJNA703972/). The amplicon 16S rRNA raw reads generated in this study have been deposited in the BioProject database under accession code PRJNA1034039 (https://www.ncbi.nlm.nih.gov/bioproject/PRJNA1034039/). The nitrogen fixation rates generated in this study have been deposited in a Zenodo repository under accession code 7883423 https://doi.org/10.5281/zenodo.7883423 (Kostka Lab, 2023). All accompanying metadata generated in this study are provided in the Supplementary Data Files and the Zenodo repository (Kostka Lab, 2023).

Research involving human participants, their data, or biological material

Policy information about studies with human participants or human data. See also policy information about sex, gender (identity/presentation), and sexual orientation and race, ethnicity and racism.

Reporting on sex and gender	This study did not have human participants
Reporting on race, ethnicity, or other socially relevant groupings	This study did not have human participants
Population characteristics	This study did not have human participants
Recruitment	This study did not have human participants
Ethics oversight	This study did not have human participants

Note that full information on the approval of the study protocol must also be provided in the manuscript.

Field-specific reporting

Please select the one below that is the best fit for your research. If you are not sure, read the appropriate sections before making your selection.

☐ Life sciences ☐ Behavioural & social sciences ☒ Ecological, evolutionary & environmental sciences

For a reference copy of the document with all sections, see nature.com/documents/nr-reporting-summary-flat.pdf

Ecological, evolutionary & environmental sciences study design

All studies must disclose on these points even when the disclosure is negative.

Study description	Study aimed to recover genomes, gene expression, and activity of microbial community associated with salt marsh ecosystems at two contrasting environmental conditions and three compartments: sediment, rhizosphere and root. Microbial communities associated with two contrasting phenotypes of Spartina alterniflora retrieved from a pristine salt marsh ecosystem in the state of Georgia, USA. A multi-omics approach was employed to interrogate the microbial communities from 4 tall and 4 short phenotype plants, rhizosphere and sediment compartments (total = 24 samples for metaG and 16 samples for metaT). In addition, from the

same Spartina phenotypes and compartments, 5 independent samples were used to calculate rates of nitrogen fixation and stable isotope analysis.

Research sample	Our research sample are the microbial communities associated with the root, rhizosphere and sediment compartments of Spartina alterniflora. Our samples represents an area of 2.6 hectares in a Georgia barrier island.
Sampling strategy	No formal sample size calculations were performed. Samples sizes were chosen based on standards for the field of study. Specifically, four transects were established in a 2.6 hectares area in Sapelo Island, GA. About 100 meter transects were established along environmental stress gradients on salinity, anoxia and sulfide toxicity. Plants were retrieved from the end extremes of the established transects, collecting one plant per sampling point (total sampling points = 8) for shotgun metagenomic analysis. Three compartments were studied: sediment, rhizosphere and root.<br>For rate measurements and metatranscriptomic analysis the end extreme of one transect was selected since the microbial composition and microbial rate measurements and sediment and porewater biogeochemistry were similar for the four assessed transects (Rolando et al., 2020). Five independent plants at least 3 meters apart were sampled at the two Spartina alterniflora extremes for rate measurements, while four independent plants were sampled for metatranscriptomic analysis.
Data collection	Metagenomic analysis was performed from bulk sediment, rhizosphere and root samples of 8 Spartina alterniflora plants in Sapelo Island, GA. Metatranscriptomic analysis was performed only for bulk sediment and root samples due to limited rhizosphere samples.<br>Field sampling was performed by Jose Luis Rolando, Max Kolton, Tianze Song, Yutong Liu and Joel Kostka.<br>Next generation sequencing was performed at the Georgia Tech Sequencing Core using Illumina's NovaSeq 6000 System.<br>Stable isotope analysis from enriched samples were sent to analysis to the University of Georgia Center for Applied Isotope Studies laboratory. Quantitative PCR and RT-qPCR was collected using the StepOnePlus platform by Jose Luis Rolando (Applied Biosystems, Foster City, CA, USA).
Timing and spatial scale	Analysis were performed during a time lapse of three years. All sampling efforts were performed during the month of July. Thus, characterizing mid-summer conditions. The four studied transects were distributed along an area of 2.6 hectares in a salt marsh ecosystem of Georgia, USA.
Data exclusions	No data was excluded
Reproducibility	All used software is open-source and all generated data and custom scripts are available on-line for reproduction of performed analyses. GPS points of sampling locations, details of sample processing and analysis are provided in materials and methods to allow reproduction of our study.
Randomization	Rate measurements: No randomization was applied since experimental conditions were performed in the exact same conditions and same time for all analyzed samples.<br>Multi-omics analysis: Sampling and processing was performed exactly the same for all sampled plants. Sampling of contrasting plants was performed the same day and time for all transects. No randomization was necessary.
Blinding	No blinding was performed. Blinding was not required as the all performed analysis are quantitative and did not require subjective judgment or interpretation.

Did the study involve field work? ☒ Yes ☐ No

Field work, collection and transport

Field conditions	Field sampling was performed in mid-July of years 2018, 2019, and 2020 in Sapelo Island, GA. Sampling was performed at a low-elevation intertidal salt marsh ecosystem. Average annual precipitation in Sapelo Island is 1,269 mm with about half of the precipitation occurring between June and September. Annual average maximum, mean and minimum daily temperature is 25.1 °C, 20.2 °C, and 15.3 °C, respectively. In July, mean daily maximum, mean and minimum temperature is: 32.9 °C, 28.3 °C and 23.8 °C, respectively.
Location	Location in degrees:<br>ID Latitude Longitude<br>T1-P1 31.38894 -81.27755<br>T1-P3 31.38943 -81.27757<br>T2-P1 31.38866 -81.27693<br>T2-P3 31.38930 -81.27705<br>T3-P1 31.38892 -81.27632<br>T3-P3 31.38957 -81.27657<br>T4-P1 31.38904 -81.27569<br>T4-P3 31.39001 -81.2758
Access & import/export	All sampling was performed within the United States in the state of Georgia. Sampling was performed with authorization from the Georgia Department of Natural Resources (File: LOP20190067).
Disturbance	Only disturbance performed to the ecosystem was trampling and collection of limited plants for incubations and molecular work.

Reporting for specific materials, systems and methods

We require information from authors about some types of materials, experimental systems and methods used in many studies. Here, indicate whether each material, system or method listed is relevant to your study. If you are not sure if a list item applies to your research, read the appropriate section before selecting a response.

Materials & experimental systems

n/a	Involved in the study
☒	☐ Antibodies
☒	☐ Eukaryotic cell lines
☒	☐ Palaeontology and archaeology
☒	☐ Animals and other organisms
☒	☐ Clinical data
☒	☐ Dual use research of concern
☐	☒ Plants

Methods

n/a	Involved in the study
☒	☐ ChIP-seq
☒	☐ Flow cytometry
☒	☐ MRI-based neuroimaging
